# Lessons From the Past, Hope for the Future: A Qualitative Study on the Lives of Leprosy-Affected Residents of a Leprosy Settlement in Malaysia

**DOI:** 10.1177/10497323251321727

**Published:** 2025-04-22

**Authors:** Norana Abdul Rahman, Vaikunthan Rajaratnam, Noor Hanis M. Rafee, Cynthia Ramachandran, Ruth M. H. Peters, Karen Morgan, Mohamed Rusli Abdullah, Marjolein B. M. Zweekhorst

**Affiliations:** 1 Centre for Research Excellence, 263878 Perdana University, Serdang, Malaysia; 2 Athena Institute, 375153 Vrije University, Amsterdam, Netherlands; 3 Department of Orthopaedic Surgery, 150819 Khoo Teck Puat Hospital, Singapore; 4 National Leprosy Control Centre, 161785 Hospital Sungai Buloh, Sungai Buloh, Malaysia; 5 School of Medicine, 26696 RCSI-UCD Malaysia Campus, Georgetown, Malaysia; 6 School of Medical Sciences, Universiti Sains Malaysia, Kubang Kerian, Malaysia

**Keywords:** leprosy, Hansen’s disease, qualitative study, lived experiences, Sungai Buloh Leprosarium, Malaysia

## Abstract

The Sungai Buloh Leprosarium in Malaysia, established in 1930, provided relief for many individuals with leprosy, yet their personal narratives remain largely untold. This study explored the lived experiences of older individuals affected by leprosy at the Leprosarium, focusing on their concerns, needs, and challenges. By examining the historical context and key themes from interviews with leprosy-affected participants, the study aimed to enhance their well-being and provide insights applicable to leprosy-affected individuals in the community, other leprosaria, and older individuals in institutional care settings. The participants comprised eight women and ten men, aged 41–84 years, with a mean age of 73.7 years. These participants, selected via purposive and snowball sampling, were interviewed for 40–45 minutes over two sessions to allow rest breaks. The interviews were transcribed, validated, and analyzed using NVivo 14 software through an inductive–deductive thematic approach. Results showed hope is a transformative coping strategy, emerging as the overarching theme, guiding participants through the challenges of leprosy. Other themes included navigating the bio-psychosocial challenges of leprosy, life within the leprosarium, practical coping strategies in daily life, parental separation from infants at birth and its emotional toll, and expressing gratitude for the opportunities and care provided by the leprosarium. These themes demonstrated how hope shaped participants’ resilience and resourcefulness, enabling them to positively reframe their experiences. This study emphasized the role of hope and the importance of support systems in fostering resilience and improving the quality of life of individuals with leprosy and older adults in institutional care settings.

## Introduction

Leprosy, or Hansen’s disease, caused by *Mycobacterium leprae*, is one of the oldest known infectious diseases, impacting societies globally for centuries (Bennett et al., 2008; Santacroce et al., 2021). Leprosy targets the skin and nerves, causing significant physical impairments if left untreated. It is classified into paucibacillary (PB) and multibacillary (MB) leprosy (WHO, 2023). PB leprosy is milder, featuring five or fewer skin lesions without bacilli in skin smears, while MB leprosy is more severe, with more than five skin lesions, nerve involvement, or bacilli present in the slit-skin smear (WHO, 2023). The debilitating effects of leprosy and visible deformities fostered prejudice, fear, and segregation, resulting in the social isolation and poverty of affected individuals (Bennett et al., 2008; Santacroce et al., 2021). These conditions were worse in ancient times when there were no effective treatments (Santacroce et al., 2021). The introduction of multi-drug therapy (MDT) in the 1980s has reduced the global prevalence, with the burden now primarily affecting Asia, South America, and Africa (Bennett et al., 2008; Smith et al., 2017). Understanding the historical context of leprosy sheds light on the persistent stigma surrounding the disease despite advancements in medical treatment (Bennett et al., 2008; Santacroce et al., 2021).

Historically, due to fears of contagion, limited understanding of disease transmission, and the lack of effective drugs to treat leprosy, individuals with leprosy were isolated to leprosy colonies established by governments in geographically remote areas, removed from the general public, often on islands (International Leprosy Association—History of Leprosy, n.d.a; Inglis, 2014; Kaehler et al., 2015; Lesshsafft et al., 2010; Sato & Frantz, 2005). Some colonies began as squatter settlements where affected individuals sought refuge together (Staples, 2014). Over the years, these colonies have played a vital role in providing care and a sense of identity and belonging to the residents (Staples, 2014). Studies indicate that residents of leprosy colonies often have better access to general and specific healthcare schemes compared to the general community (Monickaraj & Moturu, 2019; Staples, 2014). Although many colonies have closed, some still exist not as isolation facilities but as communities where people live together (Staples, 2014; The Leprosy Mission, n.d.). The United Nations reported that up to 2000 leprosy colonies remain globally (Demopoulos, 2023; United Nations, 2023), particularly in countries like India (Sen, 2023), China (Shen et al., 2007; Staff, 2019), Nepal (Ratcliffe, 2019), Brazil (Nolen, 2015), and Nigeria (Akinfenwa, 2023; Salako, 2022).

The Sungai Buloh Leprosarium in Malaysia was established in 1930 as part of colonial public health measures to control leprosy (Federated Malay States. Medical & Health Services, 1933; Joshua-Raghavar, 1983). It exemplified the dynamic evolution of leprosy management from isolation to a community-focused approach (Joshua-Raghavar, 1983; UNESCO World HeritageCentre, 2019). Designed as a self-sufficient community, the Leprosarium provided essential amenities but also imposed strict segregation, limiting interactions with the outside world (Joshua-Raghavar, 1983). Despite being separated from their families and communities, this setup allowed residents to foster a sense of normalcy and community within the settlement. However, physical and social barriers perpetuated leprosy-related stigma (Joshua-Raghavar, 1983; Loh, 2009).

Despite its historical significance, research on the lived experiences of the Sungai Buloh residents is limited, with most studies focusing on clinical treatments (Pearson et al., 1975; Pettit & Rees, 1964; Reddy & Raju, 2006; Travers, 1926; Waters et al., 1978) rather than the socio-cultural (Nery et al., 2019) and psychological impacts (Somar et al., 2020). This study seeks to bridge this gap by examining narratives, social interactions, and perceptions within their historical and cultural contexts (Dahlstrom, 2014), from their initial admission to the Leprosarium through to their current circumstances. These individuals are now free to leave but prefer to remain in the settlement because they find comfort in being surrounded by people with similar backgrounds and experiences. Understanding these challenges is important for developing targeted interventions and support systems (O’Cathain et al., 2019), tailored for this ageing population.

This study explored the lived experiences of older individuals affected by leprosy at the Leprosarium, focusing on their concerns, needs, and challenges. By examining the historical context and key themes from interviews with leprosy-affected participants, the study aimed to enhance their well-being and provide insights applicable to leprosy-affected individuals in the community, other leprosaria, and older individuals in institutional care settings. These findings hold relevance for policymakers, practitioners, and service providers, contributing to greater inclusivity and support within such environments.

## The Sungai Buloh Leprosarium

The Sungai Buloh Leprosarium (Federated Malay States. Medical & Health Services, 1933; Joshua-Raghavar, 1983; Loh, 2009) was established 4 years after the introduction of the 1926 Leper Enactment Act in Malaya (Jayalakshmi, 1994; Singapore Legislation, n.d.), which mandated the compulsory notification and isolation of leprosy patients. It became the largest and most advanced leprosarium in the British Commonwealth and a leading leprosy research center (Joshua-Raghavar, 1983, pp. 87–96; Pettit & Rees, 1964; Travers, 1926; Waters et al., 1978). Located near Kuala Lumpur, the capital of Malaysia (Wikipedia, n.d.), locally known as the “Valley of Hope,” it was designed by the British as a self-sufficient community that provided medical care to leprosy-affected residents from various parts of Malaysia and neighboring countries (Joshua-Raghavar, 1983). While some residents were brought involuntarily, many came on their own accord to seek treatment at the Leprosarium (Joshua-Raghavar, 1983, pp. 60–61; Phang & Wong, 2006). It surpassed other leprosy colonies in Peninsular Malaysia and was comparable internationally to facilities like the Culion Leprosarium in the Philippines (International Leprosy Association—History of Leprosy, n.d.a; Republic of the Philippines, Department of Health, 1956), known for its self-contained living conditions.

The Leprosarium provided residents with educational opportunities, skills training, job opportunities, and engaging social and recreational activities (Joshua-Raghavar, 1983). These initiatives fostered meaningful relationships among the residents and nurtured a strong sense of belonging to the community (Care & Share Circle, n.d.; Joshua-Raghavar, 1983, pp. 139, 149; Loh, 2009; Phang & Wong, 2006; Sasakawa, 2023, p. 73; Tan et al., 2012). Residents were encouraged to take up agriculture and farming in designated green areas (Care & Share Circle, n.d.; Joshua-Raghavar, 1983, p. 81; Sasakawa, 2023, p. 73). The horticultural industry they started served as the economic foundation and distinctive characteristic of the Leprosarium until today (Care & Share Circle, n.d.; Joshua-Raghavar, 1983, p. 148; Loh, 2009). Volunteers continue to offer regular arts and crafts activities and cultural trips to engage and maintain a sense of community for those remaining at the settlement (Care & Share Circle, n.d.).

In the early years of the Leprosarium, physical disabilities among residents were common due to the experimental nature of leprosy treatments (Federated Malay States. Medical & Health Services, 1933; Joshua-Raghavar, 1983). Before antibiotics, various remedies were tested, including a traditional Chinese medicine called Tai Foong Chee, derived from chaulmoogra nuts, which offered some hope but yielded varying results (Muir et al., 1928; Souze-Araujo, 1960; Travers, 1926). The discovery of sulfone therapy (Promin) in the 1940s marked a breakthrough, and efforts to reduce treatment toxicity led to the use of Dapsone, the parent compound of Promin, as standard long-term monotherapy until drug resistance emerged in the 1970s. The Leprosarium played a key role in the late 1940s trials of Dapsone and alternative leprosy drugs (International Leprosy Association—History of Leprosy, n.d.b; Joshua-Raghavar, 1983). Sungai Buloh Leprosarium significantly contributed to the development of the morphological index and drug resistance investigations. In 1964–1965, in collaboration with the British Medical Research Institute, researchers provided definitive proof of Dapsone resistance in three patients using the mouse footpad inoculation method. The first case of primary resistance was detected in 1976 (International Leprosy Association—History of Leprosy, n.d.b; Joshua-Raghavar, 1983; Pearson et al., 1975; Pettit & Rees, 1964). The MDT was piloted at the Leprosarium in 1985 before nationwide implementation.

The residents of the Leprosarium were not allowed to mix between men and women until they were married (Care & Share Circle, n.d.; Joshua-Raghavar, 1983). While married couples were allowed to have children, their infants were separated at birth as a precaution against transmission of the infection. The Baby Home cared for these infants for 6 months (Care & Share Circle, n.d.; Joshua-Raghavar, 1983). Similar practices were observed in other leprosaria, such as the Culion Leprosarium in the Philippines (Republic of the Philippines, Department of Health, 1956). At the Sungai Buloh Leprosarium, parents were offered the choice to arrange for childcare or to place their children for adoption after 6 months (Care & Share Circle, n.d.; Joshua-Raghavar, 1983).

The 1969 National Leprosy Control Programme was another cornerstone in leprosy history in Malaysia, which integrated leprosy treatment into general medical services (Jayalakshmi, 1994; Joshua-Raghavar, 1983, pp. 126–131; Kamaludin, 1990). This integration eliminated the need for segregation following the repeal of the 1926 Leper Enactment Act (Singapore Legislation, n.d.). Even after being cured and given the option to integrate into the larger community, many residents hesitated to leave the settlement due to a lack of alternative living arrangements and the persistent fear of discrimination (Joshua-Raghavar, 1983; Loh, 2009; Phang & Wong, 2006). Some who left returned after experiencing life outside its confines (Joshua-Raghavar, 1983; Loh, 2009; Phang & Wong, 2006).

At its peak in 1957, the Leprosarium housed 2435 patients (Joshua-Raghavar, 1983; K. G. Lim, 2016; Loh, 2009). As of 2023, it accommodates 99 older residents (Care & Share Circle, n.d.), all above 60 years old, who have completed their treatment, and one temporary resident, a 41-year-old currently undergoing treatment. The hospital authority occasionally admits individuals temporarily to provide essential care and support. Most residents at the Sungai Buloh Leprosarium live independently in chalets within the settlement, while those needing more care are housed in hospital wards (Care & Share Circle, n.d.). These residents continue to receive support from the government and various organizations to manage the long-term effects of leprosy (Care & Share Circle, n.d.; Loh, 2009; MaLRA, n.d.; Phang & Wong, 2006).

In recent years, the Leprosarium has undergone significant transformations, converting a section of the settlement into a General Hospital and university facilities while retaining the living accommodations for former leprosy patients (Care & Share Circle, n.d.; Y. L. Lim, 2015; Loh, 2009, 2011).

### Materials and Methods

The methods section outlines the research design and data collection strategies used to explore the experiences of leprosy-affected individuals at the Sungai Buloh Leprosarium.

### Study Design

The research adopted a qualitative approach, conducting in-depth, face-to-face interviews guided by a literature-informed interview guide (Appendix I). A case study approach was selected to explore the experiences of leprosy-affected individuals at the Sungai Buloh Leprosarium. This method provided an in-depth understanding of participants’ historical and present-day experiences within the unique setting of the leprosarium and insights into broader systemic factors influencing their lives.

### Study Area

Sungai Buloh Leprosarium is the only remaining leprosarium in Malaysia. It serves as a key study site, chosen for its unique status, providing a wealth of historical data with residents who have experienced life in the settlement before effective treatments were available. These factors make the Leprosarium invaluable for studying the long-term impacts of leprosy on their lives and the broader issues of health, stigma, and community involvement. This study is part of a larger research endeavor encompassing three distinct study locations in Peninsular Malaysia, highlighting the Leprosarium’s significance within the wider context of leprosy research in the region.

### Participants

Our initial recruitment target was 12 participants per study location, informed by the findings of Guest et al. (2006) and Ando et al. (2014), which indicated that saturation generally occurs after 12 interviews. We were prepared to increase the number of participants if necessary. The primary researcher, NAR, communicated and met the doctors overseeing the Sungai Buloh Leprosarium and the leaders of the Sungai Buloh Settlement Council, who played a key role in helping to identify residents open to participating in the study and possessing effective communication skills. Participant selection was primarily through purposive sampling and some by a snowball approach.

Efforts were made to maintain gender balance and included individuals across different stages of leprosy (WHO, 2023), including those with visible disabilities (grade 2 disability), like deformities of their hands and feet, facial disfigurements, and blindness (Brandsma & Van Brakel, 2003). Initially, nine women and nine men volunteered; however, one woman withdrew and was replaced by a male participant. Thus, eighteen participants, consisting of eight women and ten men, aged 41–84 years, with a mean age of 73.7 years, were recruited. All participants could provide written informed consent. Every volunteer was included to respect their willingness to contribute to the research and value their unique perspectives and experiences. Additionally, it would enhance the study’s validity and potentially achieve data saturation. This approach reflects a commitment to inclusivity and honoring diverse perspectives within the Sungai Buloh community.

### Interview Guide

Two researchers (NAR and VR) developed an interview guide (Appendix I) based on the literature and guided by Engel’s biopsychosocial model (Engel, 1977). The guide would ensure consistency across discussions. It featured open-ended questions designed to encourage participants to share their stories, describing their experiences and current life situations, to understand their concerns about leprosy, their identified needs for support and care, particularly as they are older, and the challenges faced during emergencies living at the settlement. The interview sought to unravel the emotional, social, and practical aspects of their lives, recognizing the complex factors that influence their well-being. The guide sought to uncover nuanced insights into the participants’ relationships within both the Leprosarium and the broader community, shedding light on the social dynamics and support networks. Probing questions were thoughtfully included to encourage elaboration and to ensure depth and richness in the gathered information.

### Data Collection

NAR met the potential participants at the Central Clinic, the main outpatient facility for the residents. After providing comprehensive study details, participants were encouraged to ask questions and assured of confidentiality and the right to withdraw without consequences. Written informed consent was obtained when participants were ready.

The interviews were conducted between 1st and 30th November 2022 at locations chosen by the participants within their familiar environment, including the Central Clinic, the Settlement Council office, their homes, the local café, and the hospital wards.

Each interview session lasted 40–45 minutes, with most participants interviewed at least twice, although some extended beyond the allotted time. They were allowed to take breaks if they felt tired. All the interviews were audio-recorded, with the participants’ consent. Some participants chose to have a trusted friend present, while others brought photographs to enrich their accounts. After each interview, a brief verbal summary was shared, outlining the main points and significant insights shared by the participants.

### Data Processing and Analysis

All interviews were transcribed verbatim to create a comprehensive record. The participants were invited to verify the accuracy of these transcriptions. The analysis was conducted using NVivo 14 software, employing inductive and deductive thematic analysis, integrating predetermined categories and emergent themes from the data. Two coders (NAR and VR) carefully read the transcriptions and identified recurring themes and patterns. They noted alignment between some themes and those found in the literature while also identifying new context-specific themes and categories. Discrepancies in the coders’ interpretations were resolved through discussion, aided by memo writing and annotations. This rigorous approach ensured clarity and consistency in data analysis.

### Trustworthiness and Rigor

To ensure the trustworthiness of the study, we employed triangulation using multiple data sources by comparing data from interviews, field observations, and historical records. We also conducted member checks, where participants reviewed and confirmed their interview transcripts. The primary researcher (NAR) also built trust by attending and participating in some of the activities at the Leprosarium. Two coders (NAR and VR) independently analyzed the data, with discrepancies resolved through discussion. An audit trail documenting decision-making and reflections on data collection and analysis was maintained. The study provided contextual details about the participants and settings to facilitate an understanding of the research environment and its potential applicability to similar contexts.

### Ethical Approval

This study received ethical approval from the Institutional Review Board of Perdana University, Malaysia (PUIRBHR0321(B)) and the Medical Research Ethics Committee of the Ministry of Health Malaysia (NMRR ID-22-00442-GNI (IIR)). The primary researcher (NAR) obtained written informed consent from all participants, and despite its voluntary nature, no one chose to drop out. All identifiable information was anonymized to maintain confidentiality. The data was kept securely in a password- and biometric-protected laptop.

### Researcher’s Positionality

As a researcher fluent in the local languages and familiar with the culture, NAR has a unique perspective on this study. She studied at a local university and was exposed to the Sungai Buloh Leprosarium as a medical student in the 1980s, participating in clinical rotations and observing care practices firsthand. This early exposure has given her a historical perspective on the leprosarium and its evolution over the decades. She has conducted research on leprosy in Indonesia and engaged in activities at the Sungai Buloh settlement, building trust with the community. She is aware of her biases and power dynamics, practising reflexivity and ensuring ethical standards. Her background has helped to accurately capture the lived experiences of the residents, providing valuable insights.

The second researcher (VR) is a surgeon with experience working with marginalized communities, including individuals with Hansen’s disease and disabilities. VR engaged in ongoing reflexivity throughout the research process by regularly discussing interpretations with NAR to minimize biases and ensure that the data analysis remained grounded in the participants’ voices.

## Results

This section outlines the demographic details (Appendix II) and insights from interviews with leprosy-affected residents of the Sungai Buloh Leprosarium, aiming to understand their lived experiences.

## Demography and Characteristics of Participants

The participants comprise eight women and ten men, aged 41–84 years, with a mean age of 73.7 years. The group includes thirteen Chinese, five Malays from various states of Peninsular Malaysia, and one Indonesian. The youngest age at admission, dating back 5–6 decades, was 8 years old, with an average admission age of 14.9 years. Marital status varied, with 12 participants having children. Educational attainment showed three participants with no formal education, one has a professional diploma, and most having reached secondary education. The participants held various medical, administrative, and general roles at the Leprosarium, but only two are currently employed. Eleven participants live independently or with their spouses in chalets on the grounds, while seven are cared for in the hospital wards.

Residents of the leprosarium are provided with free housing and utilities. Twelve participants retained their “Papan,” a Malay term for “wooden board,” which serves as permanent identification and proof of residency at the leprosarium, containing a complete record of personal and medical information. This document is essential for accessing government support and financial benefits from the Malaysian Leprosy Relief Association (MaLRA). Four participants have previously been discharged, forfeiting these benefits, but allowed to return to live at the leprosarium, and three managed to secure alternate financial support from the Social Welfare Department. Two others were not residents but were given permission to be cared for in the ward. Five participants still receive a settlement workforce pension, and one receives a full government pension.

Two participants had the less severe PB leprosy, while sixteen had the more severe MB form, with ten exhibiting grade 2 disability, marked by visible deformities and impairments, including facial palsy, clawed hands and feet, and blindness. Currently, one participant is under supervised treatment, with the others having completed their treatment. Two participants who experienced a relapse in 2021 have since completed further treatment. All participants undergo annual monitoring by the hospital authority for signs of relapse.

## The Lived Experiences of Leprosy-Affected Residents of the Sungai Buloh Leprosarium

**Figure 1.**
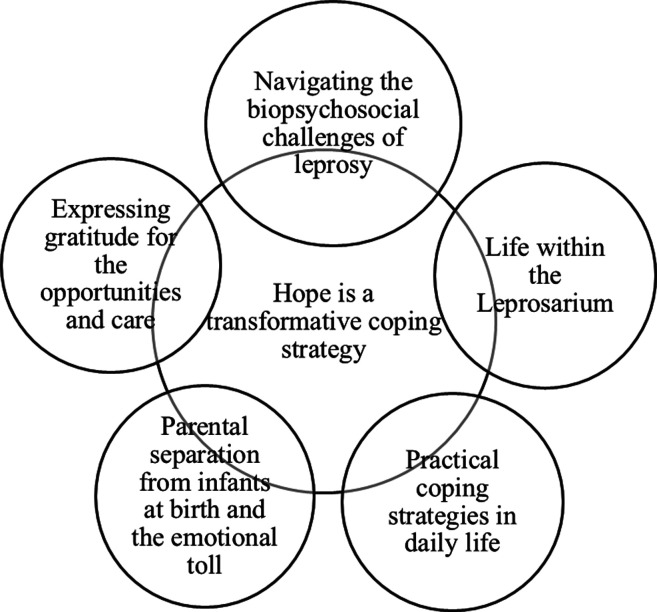
Themes emerging from interviews with individuals affected by leprosy at the Sungai Buloh Leprosarium.

Hope is a transformative coping strategy that emerged as the overarching theme in this study, guiding participants through the challenges and stigma of leprosy. Hope enabled them to endure hardships, develop resilience, and adapt and rebuild their lives after diagnosis. Participants’ narratives revealed hope intersected with specific challenges, organized into five additional themes: navigating the biopsychosocial challenges of leprosy, life within the leprosarium, practical coping strategies in daily life, parental separation from infants at birth and the emotional toll, and expressing gratitude for the opportunities and care provided by the leprosarium. These themes reflected participants’ resilience and resourcefulness in positively reframing their experiences. This section presents an overview of their experiences, organized into six themes and complemented by illustrations (Figure 1).

### Theme 1: Hope Is a Transformative Coping Strategy

This overarching theme captured how participants navigated the complex challenges of leprosy through transformative strategies. Central to this theme is hope, or “harapan” in Malay, which served as the lens through which participants interpreted their circumstances. Hope fostered resilience, enabling them to reframe their experiences, focus on long-term aspirations, and adapt to adversity. While participants expressed hope differently, they shared a determination to overcome and be cured of leprosy, find comfort and meaning within the community, and face the long-term emotional toll of family separation, stigma, and isolation. It motivated them to seek treatment, endure painful interventions, and sustain their drive to persevere. These quotes illustrate their determination:

When I discovered I had leprosy at 14, I immediately came here. My family felt my chances of getting treated were better here. I travelled by boat from Indonesia with a friend. The doctors here were mostly Europeans. I considered myself fortunate that Sungai Buloh welcomed me. (S7, male, 70–80)

My father knew I had leprosy, and we pleaded with doctors to send me here. It was only after consulting with a third doctor that I received a letter for admission. After some tests, I received the treatment that cured me. I took the medication for a very long time. (S8, male, >80)

I came to the leprosarium hoping to be cured, and here, I found friends who accepted me. (S6, male 70–80)

Before being admitted here, I couldn’t afford to travel to the treatment centre. I was informed that Sungai Buloh Leprosarium is a special hospital, and I am grateful to be admitted to the ward to facilitate my treatment. I am hopeful I will recover and return to my work as a rice farmer. (S9, male, <70)

### Theme 2: Navigating the Biopsychosocial Challenges of Leprosy

This theme showed how participants navigated the multidimensional impacts of leprosy by confronting physical, psychological, and social challenges. Through hope, social connections, and purposeful engagement, they transformed their challenges into resilience and opportunities.

Leprosy exerted a significant biophysical impact on the affected individuals, manifesting as skin changes, nerve pain, and deformities. The experimental treatments, which were less effective than modern MDT, contributed to the disfigurements and deformities among these individuals. Despite the severe side effects of the experimental drugs and the accompanying pain and suffering, participants were motivated to endure the challenging treatment regimens because they wanted to be cured. Their resilience in the face of physical changes showed their strength and ability to adapt to the evolving nature of leprosy treatment until an effective treatment was discovered. Below, S12’s story shows the persistence of hope and his determination to be cured despite earlier feelings of rejection and stigma. S3 further illustrated the emotional toll of chronic nerve pain and the harsh effects of treatment.

I had skin lesions—raised, red patches on my arms and face. I also lost my eyebrows. The rashes would get bright red in the sun, and my schoolmates would laugh at me. As a result, I dropped out of school. I went for Chinese medicine and consulted temple mediums, but it did not get better until I came here and was treated with Dapsone injections. My skin broke down very badly, but I stuck with the treatment. My skin slowly improved, and now, I only have some faint marks on my arms. (S12, male, 70–80)

I had no sensation in my legs initially, and while the deformities were less severe then, compared to now, the nerve pain was excruciating. I cried in pain every night. Despite the severe side effects of the treatment, I endured them because they relieved the pain in my legs. (S3, female, >80)

Participants revealed at the time the Sungai Buloh Leprosarium was established, there was a palpable societal fear of leprosy, and a forced segregation policy was in place in Malaya. Our interviews uncovered a significant lack of awareness and knowledge about leprosy among participants before their admission to the Leprosarium. Many were too young to understand the disease, which was often feared and misunderstood in their communities. Participants avoided hospitals and resisted admission to the Leprosarium because they feared being separated from their families. Various factors, including geography, economic constraints, lack of awareness, and cultural barriers, influenced their access to leprosy treatment. The following quotes show how limited awareness and knowledge about leprosy delayed early diagnosis and exacerbated fear:

Those years ago, I hid at home to avoid police detention after developing leprosy symptoms. When my condition worsened, my uncle brought me to the General Hospital, where I was diagnosed with leprosy. The doctor gave me a letter for admission to Sungai Buloh, but I continued to hide at home. One day, my uncle deceived me by taking me to the Leprosarium and leaving me at their doorstep. I was twelve years old. He feared my family would be penalized because of me. (S15, female, 70–80)

The problems I faced when I was diagnosed in 2022 were quite similar to what other residents here experienced decades ago. I delayed consulting a doctor because I did not know I had leprosy, and when I did seek help, the doctor mistook it for an allergy. My treatment was further delayed because the nearest treatment center was too far from home, and I couldn’t afford the travel costs. (S9, male, <70)

I had a deformity of my hand since I was a little boy, and all the villagers thought I was born with it. It slowly worsened, and I had pain that I went to check at the hospital. It took repeated visits because even the doctor did not know it was leprosy. If my father had known about it, he would have taken me earlier to the hospital. (S11, male, 70–80)

Participants spoke about the rupture of familial bonds when admitted to the leprosarium. They were separated from their loved ones, stemming from the stigma and social discrimination surrounding leprosy. Many came from large, impoverished families, compounding their challenges. These illustrations show the challenges they faced and how new friendships made family separation more bearable:

My marriage broke up, and I had to ask my adopted mother to look after my little girl when I was admitted to the leprosarium. (S18, female, 70–80)

I come from a very poor family; my parents could not afford to visit me at Sungai Buloh […] I told them they did not have to visit because I was happy here. I had many friends. When I started working, I saved my money so I could go home to visit my family at Chinese New Year. (S12, male, 70–80)

My father reluctantly sent me here so I could get treated. I cried every day initially, but things improved as I found other children in similar situations. (S16, female, 70–80)

Psycho-affective disorders like sadness, shame, and anxiety were common since they had lost contact with loved ones. Rejection was especially hard to endure in this society that places significant importance on family and community. Yet, mental health support was not prioritized, with many choosing to manage their symptoms in their own way. These quotes illustrate their reflection on their lives and the deep emotional impact of family separation, and while dreams and memories evoke sadness, some found comfort in their present relationships:

Sometimes, I have bad dreams—I dream of my village and my family. It’s difficult for me to explain to you. I feel sad when I think of my life, but I accept it. (S10, male, >80)

I have not seen my family since I left home, and they have not tried to contact me either. I don’t think about my past. My husband has passed away, and I am just grateful that my son and his family live close by. (S2, female, 70–80)

The economic impact of leprosy on residents of the Leprosarium includes a combination of government support, assistance from non-governmental organizations (NGOs), and residents’ own initiatives to generate income. Those capable of working undertook various jobs within the settlement, earning modest allowances. These quotes describe how economic initiatives and external support helped residents maintain independence and dignity while their flower nurseries helped challenge the stigma surrounding leprosy:

The Medical Superintendent encouraged us to cultivate the land around our chalets. It kept us busy and made our surroundings beautiful. We are older now, and many have given up, but some of us still have our flower nurseries on a smaller scale. Until today, the public continues to buy our plants. (S15, female, 70–80)

We are grateful for the benefits we receive, and we are given free medical care. We are comfortable. (S4, female, 70–80)

### Theme 3: Life Within the Leprosarium

This theme explored life at the Sungai Buloh Leprosarium, which was more than a medical facility, offering residents a home-like environment with amenities like shops, a post office, a police station, and a school. Residents, constrained by apprehensions from people outside were compelled to take on various jobs within the settlement. While they could move independently within its confines, they could not leave without permission. Despite the restrictions, the Leprosarium provided stability, a sense of community, and purposeful engagement, enabling them to build resilience, form relationships, and find meaning beyond their challenges. The experience of being institutionalized at the Sungai Buloh Leprosarium brought mixed feelings—while it offered a place to live without judgment, the strict rules created a sense of isolation and loss of autonomy.

Early regulations mandated obtaining permits for residents who wished to undertake home visits or outings beyond the Leprosarium and for their families to visit. To control infection, everything had to be sterilized before leaving, and the Leprosarium even had its own currency to prevent external circulation. These policies were designed for safety, to protect the residents and the public. Initially, many sought a cure and discharge, but they had to endure the long and complicated treatments. After so many years living in the settlement, many chose to remain, finding comfort in familiar routines and the close-knit community that provided a sense of belonging and security. The following illustrations show the impact of institutionalization—the tension between care and control, and how it fosters dependency and complicates reintegration:

The discipline here was very strict before—you would need to get permission to leave the settlement. You must give a reason for the leave and specify the number of days that you will be out. You must come back on the specific date, or they will come to find you. (S14, male, 70–80)

I struggled to adapt to life outside the settlement after spending decades here. I did not have friends or family and frequently faced inquiries and stares about my deformities. Finding employment was particularly stressful, and this difficulty was a key reason why I chose to return to the settlement. (S5, female, <70)

When I was admitted here in 2022, there was no quarantine or strict rules, and residents were free to come and go. I will stay here until I complete my treatment, although I am worried about my home in the village. I have had some visitors while I have been here, and the ward patients are friendly. I don’t feel isolated. (S9, male, <70)

### Theme 4: Practical Coping Strategies in Daily Life

This theme demonstrated participants’ resourcefulness in addressing the everyday challenges of leprosy, enabling them to adapt and build resilience through close friendships and participation in community activities like gardening, farming, and creative classes. Religion and spirituality also offered comfort and strength to the participants. As illustrated below, a participant recalled the cherished memories and strong support network formed during his formative years at the Leprosarium. Our interviews showed that despite being separated from their families, they had friends and did not feel ostracized by the community. Those who came at a young age lived in single-gender dormitories, attended school, and actively participated in sports, social activities, and after-school clubs, fostering personal growth and community bonds.

I prefer my old days here when I was at school—I had many friends. It used to be a lot of fun and full of activities. […] We had trips to the seaside and places of interest. Christmas time was very happy even though we had no money. […] We also have an in-house cinema with repeat screenings of popular films. We get to play many sports—like basketball and badminton. […] Now that I am much older, my friends have died, and it is quite lonely. (S12, male, 70–80)

Engaging in creative activities served as a valuable coping mechanism, offering a sense of purpose and emotional relief.

I enjoy the art classes, which provide me with an opportunity to explore my artistic skills. (S3, female, >80)

I took up photography as a hobby, and that made me very popular with everyone. (S17, male, >80)

Religion and spirituality also played a key role, with many finding comfort in prayer and religious services to cope with physical and emotional hardships and maintain a sense of normalcy. Coping strategies are essential for managing chronic illness and include emotional acceptance (see quote by S10 in psycho-affective disorders).

When I felt down, I would pray. It gives me the strength to go on. Even now, with my health issues, I still find peace in my prayers. (S11, male, 70–80)

I sometimes go to the temple—it’s quiet, and I feel calm there. It’s one of the ways I’ve learned to deal with everything. (S15, female, 70–80)

### Theme 5: Parental Separation From Infants at Birth and the Emotional Toll

This theme examined the emotional and practical challenges parents faced when separated from their newborns at birth, marked by feelings of loss, guilt, and longing. Despite these difficulties, some parents displayed resilience, advocating for their children’s welfare or maintaining connections through limited visits or communication.

At Sungai Buloh Leprosarium, residents were permitted to marry and establish their families within the settlement. Contraception was unavailable at the time. Twelve of our participants have children. Our interviews revealed that newborns were separated from their mothers at birth to prevent infection, with parents only permitted monthly, non-contact visits for an hour. After 6 months, parents had to arrange alternative care for their babies since the Baby Home only cared for infants up to that age. None of our participants placed their babies for adoption, but some mothers secured their discharge to care for their infants, losing their financial support, while fathers stayed to work at the Leprosarium, visiting them as often as allowed. Children raised by relatives or in foster care were allowed brief visits and eventually reunited with their parents when they were older or after the segregation law was repealed. A local NGO assisted individuals who were adopted to reconnect with their birth parents while also supporting parents who sought to reunite with children they had placed for adoption.

Participants acknowledged that the strict rules were implemented to protect the children, although no counselling was offered during those challenging times. Over the years, many individuals have independently coped with the psychological effects and inherent sadness, often viewing the admission of mental health issues as a sign of weakness. The availability of counselling services today marks significant progress in supporting their emotional well-being. The following quotes highlight the emotional complexity of parenting in the context of leprosy and institutional care, illustrating the sacrifices parents made and their pride in their children’s achievements, alongside the joy of seeing their grandchildren after missing much of their children’s lives:

It was hard to have your baby taken away soon after birth. We were only allowed a glance. But I knew we would be reunited, and my adopted mother was kind to care for my two daughters. She would bring them for brief visits when they were allowed by the hospital. I am very happy that one daughter now lives with me and the other close by. (S1, female, 70–80)

I sent my son to be cared for by the Salvation Army in Ipoh and my daughter to the Nuns at Convent Bukit Nanas. It was challenging to find a place that could accommodate both of them. My late husband and I had to work hard to cover their expenses—the fees were nominal, but it was still a lot for us. We would travel by train to Ipoh to visit my son, but when he was older, he was allowed to visit us during school holidays. My daughter was closer to home, but she passed away at 14 years old. Despite the hardship, my son excelled at school and earned a scholarship to study overseas. He is currently working in Bangkok but visits me when he can. I am proud of him. (S15, female, 70–80)

I felt sad when our babies were taken away at birth, especially for my wife, but that was the rule then. Fortunately, my mother-in-law agreed to care for our children. When the Leprosarium allowed the children to visit, she would bring them along, and those moments were always cherished. Though my wife has passed away, my children continue to visit me, especially during festivals, and they will bring my grandchildren. These visits bring me great joy. (S8, male, >80)

I lived outside the leprosarium, so when my children were born, they were not taken to the Baby Home. We were able to look after them ourselves. But my wife left me and took our oldest child. I had difficulty caring for my two younger daughters, so I sought help from the Church, and they were admitted to a boarding school. During school holidays, I would bring them home. I now live in the hospital ward. My children visit me regularly, bringing my grandchildren. I have also reunited with my oldest child. (S6, male, 70–80)

### Theme 6: Expressing Gratitude for the Opportunities and Care Provided by the Leprosarium

This theme highlighted participants’ gratitude for the education, care, support, and opportunities provided by the Leprosarium, which significantly transformed their lives. Expressing this gratitude exemplified a transformative coping strategy as they valued the healthcare interventions and personal development opportunities, including lifelong friendships. They appreciated the sense of purpose and income through work opportunities, with many taking up gardening and establishing flower nurseries. Despite enduring severe side effects from experimental treatments, they persevered in their desire to be cured.

As they age, they are grateful for the continued support from the government, MaLRA, and other NGOs. The financial aid, though modest, has been essential in meeting their basic needs. They also valued the enrichment brought by volunteers who organize engaging activities. The community structure of the Leprosarium has provided security while allowing them to maintain their autonomy, contributing significantly to their quality of life. The following quotes illustrate how the provision of basic needs by the Leprosarium created a foundation for stability and purpose and how gratitude helped participants reframe their experiences positively, fostering resilience:

I entered Sungai Buloh as a young boy and looking back; I believe I have had a reasonably good life. I consider myself fortunate—I received an education, I am still employed, I have many friends, and despite my physical deformities, I am independent! My family was so poor when I was growing up that I wouldn’t have had these opportunities. (S14, male, 70–80)

If I had not come here when I was young, I probably would have died—I come from a large family, and my father was a tofu seller. I am grateful to the leprosarium for providing food, shelter, and medical care. I was given the opportunity to develop my nursery business. (S15, female, 70–80)

I am 84 years old, and before my wife passed away, we lived in one of the chalets. We were self-sufficient and independent and received excellent care from the doctors and hospital staff. If we had been living outside the community, we might not have received the same level of care, and I might have been placed in a nursing home. I am thankful the hospital allowed me to opt for ward residence when I felt it was more suitable than in the chalet. (S8, male, >80)

Throughout these narratives, hope emerged as the thread that connected participants’ experiences. The overarching theme of transformative coping strategies demonstrated the interplay between participants, the leprosarium, and their resilience and determination in overcoming the challenges of leprosy.

### The Current Situation of the Older Residents of the Leprosarium

The elderly residents of the Leprosarium continue to navigate the complexities of ageing within their community. Their physical limitations and mobility made them more vulnerable in emergency situations, hindering their ability to respond promptly and exposing them to risks of personal harm and property damage. They emphasized the need for improved support systems to ensure their safety and well-being. Despite these difficulties, the close-knit nature of their community provides emotional support and mutual care.

In recent months, we have had severe flooding, affecting four households and causing damage to valuables and properties. This situation posed significant risks to our personal safety and well-being. It was due to our physical disabilities and poor mobility that we were unable to respond swiftly to the emergency to block the water’s entry points. (S14, male, 70–80)

Prolonged residency in the leprosarium has fostered a strong sense of community, providing a unique source of comfort. While some residents benefit from family visits, others experience loneliness as friends pass on, and they rely on the leprosarium’s social framework. Care and Share Circle, an NGO, has played a significant role in keeping these residents engaged by involving them in various activities. These initiatives help to maintain the community life within the leprosarium. The activities, which include gardening, cooking, arts and crafts activities, cultural trips, or just having tea at the local café, offer the residents opportunities for social interactions, which helps mitigate feelings of loneliness. Additionally, the volunteers also provide the residents with hairdressing services, spa days, and other similar activities. The NGO maintains an office on-site to help the residents in case of emergencies.

In the early days, we were encouraged to form clubs and associations based on our race, clanship, and religious beliefs, as well as non-ethnic-based organizations open to all. We organized social activities and promoted sports, aiming to connect members. Over time, these activities dwindled because we are all older, but an NGO has since taken an active role in organizing trips and events to keep the community engaged, with the help of many volunteers. (S13, male, >80)

Community engagement remains an essential component of social life. At their age, these participants do not need a job, but many said they would get involved in local initiatives and volunteer for causes they are passionate about. Many of them feel empowered to raise money through their artistic endeavors, contributing to community activities, including the local hospice, as illustrated here:

We are now much older, so we must find ways to cope and keep ourselves engaged. Besides gardening, I am involved in creative classes run by an NGO. Some of our artwork has been exhibited at the National Art Gallery. We have sold some pieces and are proud to donate the proceeds to local causes. (S5, female, <70)

## Discussion

Our research showed that the Sungai Buloh Leprosarium functioned as a self-sustaining and culturally diverse community, fostering unity through shared cultural events and activities (Joshua-Raghavar, 1983; UNESCO World HeritageCentre, 2019). Similar to other leprosaria, such as the Culion Leprosarium in the Philippines (International Leprosy Association—History of Leprosy, n.d.a; Republic of the Philippines, Department of Health, 1956), Sungai Buloh Leprosarium served as an isolation facility amidst the fear and stigma surrounding leprosy. This study revealed how societal attitudes toward leprosy were shaped by policies of segregation and the perception that leprosy was highly contagious and incurable. Sungai Buloh Leprosarium had a community-oriented setting, where residents engaged in routines resembling village life, reflecting a shift in leprosy management (Martos-Casado et al., 2022). This study enhanced our understanding of the historical and cultural contexts that shaped the lives of those affected by leprosy.

This research explored the lived experiences of the long-term residents at Sungai Buloh, mostly over 70, who have lived in the community for over 50 years. It focused on the effects of leprosy, encompassing the medical implications and its broader social and psychological impacts. Despite enduring significant hardships such as exposure to experimental treatments, mandatory admission to the leprosarium, separation from families, strained relationships, and separation from newborns, residents demonstrated resilience and found community support. They formed close relationships with other residents, engaged in meaningful activities, and received comprehensive care that upheld their dignity and purpose. Acknowledging the adversity and resilience of these individuals is vital in understanding their lives at Sungai Buloh Leprosarium. The findings, while centered on leprosy, enhanced our understanding of the challenges and needs of older adults living in long-term institutional care (Barreira et al., 2023). By understanding the physical, emotional, and social dimensions of their experiences, caregivers and policymakers can design tailored interventions that address medical needs and inform more empathetic and supportive care practices. This knowledge will improve care in leprosy-specific and general elderly care settings.

Hope was a central element in our findings, influencing how participants managed the challenges of leprosy. Participants demonstrated resilience and resourcefulness through enduring prolonged leprosy treatments, aligning with Snyder’s Hope Theory (Snyder, 2002; Snyder et al., 2001), which emphasizes goal-setting and forward-thinking to overcome adversities. Our findings emphasized hope’s role in enhancing participants’ well-being, consistent with studies linking hope, quality of life, and social support for leprosy and chronic diseases (Mardhiyah et al., 2020; Nugraheni et al., 2023; Peters et al., 2014).

Residents of the Sungai Buloh Leprosarium benefitted across several dimensions of Maslow’s Hierarchy of Needs (Maslow, 1943). Firstly, the leprosarium met their physiological needs by providing regular meals, housing, and consistent healthcare, which they valued greatly and acknowledged might not have been available outside the community. Given their advanced age, this provision helps prevent less favorable outcomes, such as malnutrition, homelessness, and inadequate medical attention, which could have led to a decline in their overall health and well-being.

The Sungai Buloh Leprosarium provided a sheltered and stable living environment, offering continuity and security that eased the stress of living independently outside the community. The close-knit community provided mutual support, addressing the residents’ social needs and creating a sense of belonging. Our findings highlighted that the comprehensive services enabled the residents to age with dignity and comfort (Care & Share Circle, n.d.; MaLRA, n.d.). In contrast, some studies have documented inadequate care, social isolation, and poor living conditions in other leprosaria (Dako-Gyeke et al., 2017; Kaur & Van Brakel, 2002), worsening stigma, poverty, and psychological suffering. Edmond (2006) highlighted the complex history of leprosy colonies, where inadequate resources, substandard living conditions, and limited access to comprehensive medical care often failed to provide even the most basic needs, such as shelter and food. The Sungai Buloh Leprosarium, by contrast, addressed these challenges through a holistic model of care that promoted dignity, resilience, and improved quality of life.

Our participants also shared significant emotional and social challenges, including feelings of isolation and loneliness exacerbated by family separation, loss of friends as they age, and the inability to integrate into the broader society, a poignant issue in cultures where family and community connections are paramount (Rafferty, 2005). These findings highlighted the need for psychological and counselling support in treatment programs, aligning with Lazarus and Folkman’s Model (Lazarus & Folkman, 1984), which emphasizes adaptive coping strategies within communities. The Leprosarium fosters strong bonds and a sense of belonging among residents, enhancing their emotional well-being and overall psychological health through the development of lifelong friendships and mutual support networks over decades. Similarly, studies by MacLeod et al. (2016) and Jung et al. (2020) emphasized the importance of community support for the well-being of leprosy-affected individuals.

Our study revealed that participants at the Sungai Buloh Leprosarium were involved in various jobs, allowing them to move throughout the settlement and contribute to its operations (Joshua-Raghavar, 1983). Work opportunities, such as teaching, policing, gardening, and establishing flower nurseries at the Sungai Buloh Leprosarium, gave residents a sense of purpose and respect. These activities helped them maintain their self-esteem by contributing meaningfully to the community. Being recognized for their efforts reinforced their self-worth, provided them with a sense of accomplishment, and strengthened their identity within the community. This sense of belonging and pride further enhanced their emotional well-being and resilience, which countered the social stigma they faced due to leprosy.

The Leprosarium facilitated opportunities for self-actualization among its residents, particularly in the later stages of life, by providing an environment where individuals could continue to grow and find purpose despite their physical limitations. Through meaningful activities such as community work, creative pursuits, and spiritual engagement, residents could explore their interests and talents. This active involvement in meaningful pursuits contributed to a sense of achievement, enhancing their overall quality of life and well-being. Numerous studies (Camic & Chatterjee, 2013; Gray et al., 2010; Perkins et al., 2021) highlight the benefits of integrating creative and cultural activities into community routines, particularly for vulnerable groups, to promote psychological well-being and enhance community cohesion by fostering shared experiences and objectives. Similarly, Tiernan et al. (2013) demonstrated that community engagement is essential for successful ageing.

In comparison to long-term institutional care, the leprosarium offers distinct advantages in terms of community bonds, purposeful engagement, and emotional support. The strong sense of belonging and long-term relationships that have developed over decades foster a supportive environment that may be less pronounced in other institutional settings. This unique combination of care provision, community cohesion, and opportunities for personal growth highlights the leprosarium’s role in addressing the specific needs of individuals affected by leprosy and in offering valuable lessons for enhancing care practices in broader elderly care contexts.

In summary, the study highlights the resilience and community strength displayed by leprosy-affected residents in Sungai Buloh. Despite the challenges of leprosy, participants have participated in various activities to find contentment and purpose through creative pursuits and community involvement. Our findings aligned with Maslow’s Hierarchy of Needs, which states that social belonging and self-actualization contribute fundamentally to quality of life. These findings offer insights applicable to those affected by leprosy living in the community, other leprosaria globally, and older individuals in institutional care settings, emphasizing the importance of supportive environments and meaningful engagement in promoting overall well-being.

Although history may not repeat itself exactly, lessons from this study highlight the value of holistic, community-based, and person-centered care approaches. Prioritizing both physical and emotional health can improve outcomes for vulnerable populations and can be applied not just to leprosy but also to managing chronic diseases and modern care settings. Healthcare providers should emphasize mental health support, policymakers need to address the unique needs of ageing institutional populations, and public health campaigns should focus on reducing stigma to facilitate reintegration into society.

## Strengths and Limitations

The study successfully enrolled enough participants and used two coders during data analysis to enhance rigor. However, potential recall bias due to participants’ advanced age might have affected the accuracy of their recollections, and social desirability bias could have influenced their responses during interviews.

To strengthen the participants’ engagement and trust, the researcher (NAR) established rapport by participating in multiple activities at the Leprosarium. The researcher ensured clarity about the study’s goals by using a multilingual approach for interviews and verifying transcriptions with participants, boosting their confidence in the research process.

## Further Research

Global comparative studies across various leprosaria could reveal resilience strategies and coping mechanisms in different socio-cultural settings. Exploring the effects of social and policy changes, psychosocial interventions, and technological innovations like telemedicine can transform care and support. Ethnographic studies would provide valuable insights into the personal and cultural realities of living with leprosy, essential for developing effective and culturally sensitive support systems.

## Conclusion

This research revealed the impact of leprosy on the long-term residents at the Sungai Buloh Leprosarium, highlighting their remarkable resilience amidst significant hardships such as experimental treatments, mandatory admissions, and family separations. The Leprosarium was not just a place of isolation but also provided a supportive community-oriented environment that provided residents with comprehensive care that addressed their physiological and safety needs while fostering strong bonds, a sense of belonging, and opportunities for self-actualization. The research emphasizes the importance of community support and tailored interventions in providing opportunities for them to lead fulfilling lives and contribute to society. The findings can be extended to the broader context of leprosy in the community and long-term institutional care, offering valuable lessons for improving the quality of life and care for older populations. The study advocates for integrating creative and cultural activities into care routines, providing psychological support, and sustaining community engagement to promote successful ageing and overall well-being.

## Supplemental Material

Supplemental Material - Lessons From the Past, Hope for the Future: A Qualitative Study on the Lives of Leprosy-Affected Residents of a Leprosy Settlement in Malaysia Supplemental Material for Lessons From the Past, Hope for the Future: A Qualitative Study on the Lives of Leprosy-Affected Residents of a Leprosy Settlement in Malaysia by Norana Abdul Rahman, Vaikunthan Rajaratnam, Noor Hanis M. Rafee, Cynthia Ramachandran, Ruth M. H. Peters, Karen Morgan, Mohamed Rusli Abdullah, and Marjolein B. M. Zweekhorst in Qualitative Health Research
