# Supplementary material for: Lessons From the Past, Hope for the Future: A Qualitative Study on the Lives of Leprosy-Affected Residents of a Leprosy Settlement in Malaysia
Source: Qual Health Res. 2025 Apr 22;36(8):805–20. doi: 10.1177/10497323251321727 (PMC13241597; doi:10.1177/10497323251321727)
Supplement: Supplemental Material - Lessons From the Past, Hope for the Future: A Qualitative Study on the Lives of Leprosy-Affected Residents of a Leprosy Settlement in Malaysia [file sj-pdf-1-qhr-10.1177_10497323251321727.pdf]

Appendix I

Interview Guide: Exploring Lived Experiences of Individuals Affected by Leprosy at Sungai Buloh Leprosarium

Introduction: Thank you for participating in this study, which aims to understand the unique experiences and challenges individuals living with leprosy face at the Sungai Buloh Leprosarium. Your insights are necessary to understand the unique challenges faced by residents. This interview aims to explore your experiences, needs, concerns, and challenges. Your openness is greatly appreciated, and confidentiality will be maintained throughout the discussion.

Background and Current Situation:

Personal background:

1. Can you tell me a little about yourself, where you are from, your family, siblings, spouse, children, occupation, etc

Experience with leprosy

2. Can you share your story about living with leprosy, describing your experiences and current life situation at the Sungai Buloh Leprosarium?

Elaborate on how you first noticed your symptoms, the age at which you first noticed them, how long you experienced them before being diagnosed with leprosy, and who you consulted?

When were you admitted to Sungai Buloh, at what age?

How did you feel when you were told to have leprosy, and how did it impact you and your family? Were any other family members affected by leprosy?

Describe your initial experiences and emotions upon arriving at Sungai Buloh, including how you coped with the separation from your family.

What treatments did you receive shortly after admission?

Did you attend school or participate in any educational or work-related activities at the settlement? What jobs did you undertake, and what were the associated benefits?

Impact and evolution

3. How has leprosy impacted your life since your admission to the Leprosarium, and how have things evolved for you over the years?

What are the most important aspects of your life right now?

Have you been able to visit your family? How did you cope with being separated from your family?

4. Reflections on the Biopsychosocial and Economic Aspects:

Physical

How has leprosy affected your physical health? Are there specific health challenges or treatments you've experienced due to leprosy?

Psychological (Psycho)

How has living with leprosy impacted your mental and emotional health? What coping strategies have you used to deal with stigma or misconceptions?

Social (Social)

How has leprosy influenced your social interactions and relationships, both within and outside the Leprosarium community? Can you describe any support networks that have helped you socially?

Economic (Economic)

How has leprosy affected your economic situation, including your employment and financial stability? Are there any economic challenges or support systems in the Leprosarium that have impacted your livelihood?

Relationships and Social Dynamics:

Relationships

5. Can you share insights into your relationships within the leprosarium and the broader community?

How do social dynamics and support networks contribute to your well-being, and are there areas where improvements could be made?

How do you handle potential stigma or misconceptions about leprosy in your interactions with the broader community outside the leprosarium?

Marriage and family

7. How has being a resident of the leprosarium influenced your perspectives on marriage and family life?

Can you describe how you navigated challenges related to marriage and family dynamics within the Leprosarium community?

How have societal perceptions and stigmas surrounding leprosy affected your family relationships?

Separation of babies

8. Can you share any insights or experiences related to the separation of babies from their mothers at the Leprosarium?

How did this separation impact your well-being and mental health? Were any efforts or support networks established to help you cope?

Concerns Regarding Leprosy:

9. Could you express your concerns regarding leprosy and its impact on your life?

What specific physical and emotional challenges have you faced due to leprosy? How has it affected your daily activities and routines?

Can you express your concerns about societal perceptions or stigma related to leprosy?

Have there been financial implications or challenges due to leprosy affecting your ability to work or engage in economic activities?

How has leprosy affected your mental well-being?

Needs for Support and Care:

11. As an older individual residing at the Sungai Buloh Leprosarium, what support and care needs do you feel are essential for your overall well-being?

What specific medical or preventive measures do you feel are essential for your well-being? Are there particular health-related needs or concerns you have as an older resident?

How do you perceive the existing support systems for older residents, and what improvements or additions would you recommend?

12. Are there particular challenges you face in accessing necessary support and care, especially considering your age?

Challenges Faced in Emergencies

13. In emergencies, what challenges do you perceive, and how do these challenges affect you within the unique context of living at the Leprosarium?

Do you believe specific measures or support systems would enhance emergency preparedness and response?

Conclusion and Additional Insights:

15. Is there anything else you'd like to share that we haven't covered in this interview? Your insights are valuable, and we welcome any further thoughts or experiences you think are important.

Closing: Thank you once again for participating in this interview. Your contributions are important in advancing our understanding and fostering support for individuals living with leprosy. If you have any further questions or concerns, please feel free to express them.

Appendix II

Demographic profile of leprosy-affected participants at the Sungai Buloh Leprosarium

Characteristics	No of participants
Gender:	
Male	10
Female	8
Age: Mean age: 73.7y	
<70	2
70-80	11
>80	5
Race:	
Chinese	13
Malay	5
Age at admission to Leprosarium	
Youngest: 8y,	
Average: 14.9y	
<12	3
12 -18	11
>18	4
Marital status:	
Single	6
Married	5
Widowed	4
Divorced	3

Children:	
Participants with children	12
Childless	6
Has children but given up for adoption	0
Education:	
No formal education	3
Primary	4
Secondary	10
Diploma	1
Occupation: ISCO-08 (ILO, 2008)	
Professional	1
Technician	1
Clerical	2
Services	4
Elementary worker	9
Unemployed	1
Living Situation	
Independent	7
With Spouse	4
In hospital ward	7
Type of leprosy	
Paucibacillary	2
Multibacillary	16
Grade 2 Disability	10
Treatment	

Completed	17
Relapsed but completed further treatment	2
Ongoing treatment	1
Resident status	
Possession of the ‘Papan’ – permanent identification record	12
Discharged but returned to live at leprosarium – ‘Papan’ and all benefits withdrawn	4
Not resident, No ‘Papan’ but permission to live in hospital	2
Income	
Food allowance + MaLRA RM1200 (Approx. 250 USD)	4
Food allowance + MaLRA + a small pension (Approx. 280 USD)	5
Ward benefits only	2
Ward benefits + government pension (Approx. 250 USD)	1
Ward benefits + private pension (Approx.300 USD)	1
Ward benefits + MaLRA (Approx 60 USD)	1
Ward benefits + MaLRA + a small pension (Approx. 100 USD)	1
Welfare financial assistance Approx. 100 USD	2
Welfare + personal earnings Approx 200 USD	1

MaLRA: Malaysian Leprosy Relief Association

Paucibacillary and Multibacillary: Refer Introduction

1 MYR = 0.21 USD (Approx.)
